# Improved k-space-based MR thermometry by joint PRF phase shift and T1/T2* attenuation estimation

**DOI:** 10.1186/2050-5736-3-S1-P19

**Published:** 2015-06-30

**Authors:** Pooja Gaur, William Grissom

**Affiliations:** 1 Vanderbilt University, Nashville, Tennessee, United States

## Background/introduction

MR temperature mapping based on the proton resonance frequency (PRF) shift is used in MR-guided focused ultrasound procedures for dosimetry and safety monitoring. While conventional PRF-shift thermometry is based on calculating a phase difference between two reconstructed MR images, Gaur et al [1,2] have recently described two algorithms that estimate temperature-induced phase shifts directly from MR k-space data, prior to image reconstruction. The approach enables large dynamic scan acceleration factors[1] and the correction of chemical-shift (CS) effects that geometrically distort the temperature maps.[2] However, that work neglected image attenuation that accompanies the PRF phase shift and is primarily caused by increasing T1 with temperature.[3] Here it is shown that attenuation degrades the accuracy of k-space-based reconstructions, but that it can be accounted for in the reconstructions.

## Methods

Simulations and experiments were performed using gradient-recalled echo scans at 3 Tesla (Philips Achieva with Sonalleve HIFU) with 16 ms echo time and 44 Hz bandwidth. A phantom was simulated with Gaussian-shaped hot spots ranging from 0 to pi and exponential attenuation factors ranging from 0 to 0.8. A tissue-mimicking gel phantom was imaged and sonicated for 41 s with a 4 mm diameter treatment cell at 110 W and 1.2 MHz. Temperature maps were reconstructed using image-domain hybrid,[4] CS-compensated,[2] and proposed joint attenuation- and CS-compensated approaches. The latter reconstructions were implemented as a refinement stage after a hybrid reconstruction. Gradient descent was used to iteratively update the temperature phase shift and exponential attenuation maps to minimize the error between the measured k-space data and the treatment k-space signal model.

## Results and conclusions

**Figure 1:**
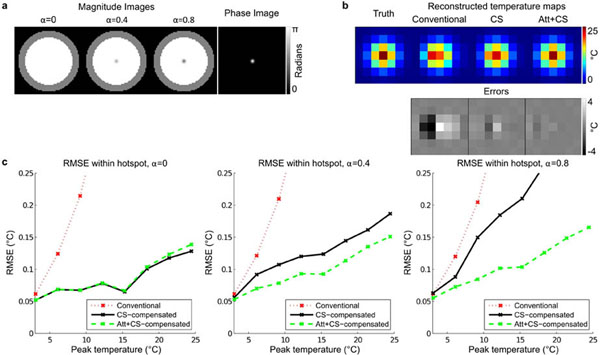
Simulation results. (a) Magnitude images with attenuation factors (alpha) and phase image. (b) Comparison of true and reconstructed temperature maps using the three algorithms. (c) Root-mean-square-error (RMSE) over the hot spot at 3 attenuation factors.

**Figure 2:**
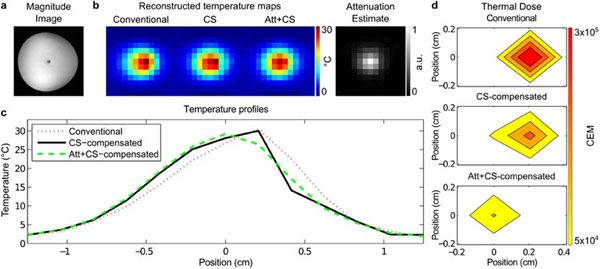
Phantom experiment results. (a) Magnitude image shows signal attenuation caused by heating. (b) Temperature reconstructions, and estimate of attenuation component. (c) Temperature profiles across the hot spot. (d) Thermal dose estimates.

Figure 1 shows the simulation results, which demonstrate that temperature reconstructions that do not account for image magnitude attenuation only partially correct chemical shift-induced geometric distortions. Figure 2 shows the phantom experiment results, in which chemical shift distortions are more completely corrected when image magnitude attenuation is accounted for, and that the small reduction of error in the temperature map for each dynamic translates into a large difference in cumulative thermal dose. These results demonstrate that accounting for image magnitude attenuations improves k-space-based temperature map reconstructions, and that it is possible to jointly estimate PRF-shift temperature maps and accompanying image magnitude attenuations using an extended k-space-based temperature reconstruction algorithm.
